# Combination of serum TBL1XR1, MFAP5, and PSA as diagnostic biomarkers for prostate cancer

**DOI:** 10.1016/j.clinsp.2026.100868

**Published:** 2026-02-09

**Authors:** Xianxian Wu, Xiuping Lin, Jiejun Lin

**Affiliations:** Ultrasound Department, Sanmen People's Hospital, Sanmen, Taizhou, Zhejiang, China

**Keywords:** Prostate cancer, PSA, Diagnosis, TBL1XR1, MFAP5, Receiver operating characteristic

## Abstract

**Objective:**

Advanced prostate cancer (PCa) remains a leading cause of cancer-related mortality in men. While Prostate-Specific Antigen (PSA)-based early screening has demonstrated mortality reduction, its diagnostic accuracy remains limited. This study aimed to evaluate the potential of serum transducin (beta)-like-1 X-linked Receptor-1 (TBL1XR1) and Microfibrillar-Associated Protein-5 (MFAP5) as novel diagnostic biomarkers for supplementing PSA-based detection.

**Method:**

Through bioinformatic analysis, TBL1XR1 and MFAP5 were identified as candidate markers, with their expression validated using reverse transcription-quantitative polymerase chain reaction. Diagnostic performance was assessed via Receiver Operating Characteristic (ROC) curve analysis, while Pearson correlation coefficients evaluated biomarker associations.

**Results:**

Serum TBL1XR1 and MFAP5 levels were elevated in PCa patients, both exhibiting positive correlations with PSA concentrations. Individual AUC values reached 0.8197 (TBL1XR1) and 0.8139 (MFAP5). The free PSA (fPSA)/total PSA (tPSA) ratio showed decreased values in PCa cases, demonstrating superior diagnostic utility compared to total PSA alone. A combined panel of TBL1XR1, MFAP5, and PSA achieved an AUC of 0.939 with a Youden index of 0.756.

**Conclusions:**

TBL1XR1 and MFAP5 have the potential for PCa diagnosis. The synergistic application of TBL1XR1, MFAP5, and PSA significantly enhances diagnostic accuracy. These findings indicate that serum TBL1XR1 and MFAP5 represent promising diagnostic markers for PCa. The combination of serum TBL1XR1 and MFAP5 with PSA for diagnosis may potentially reduce unnecessary biopsies in clinical practice.

## Highlights

• TBL1XR1 and MFAP5 are highly expressed in the serum of patients with PCa.
• TBL1XR1 and MFAP5 expression levels are positively related to PSA concentration.
• Combination of TBL1XR1, MFAP5 and PSA improves the accuracy of PCa diagnosis.

## Introduction

Prostate cancer (PCa) is a prevalent male genitourinary malignancy, accounting for approximately 7% of newly diagnosed cancers in men annually and affecting over 1.2 million individuals globally.^1^ The disease predominantly occurs in men aged over 65-years, with genetic predispositions and unhealthy lifestyle factors serving as significant risk contributors.^2^ While early-stage PCa typically exhibits favorable survival outcomes, advanced disease remains associated with poor prognosis due to high recurrence and metastasis rates.^3^^,^^4^ The serum Prostate-Specific Antigen (PSA) test remains the primary strategy for PCa early detection, having demonstrated mortality reduction benefits; however, its clinical utility is constrained by limited diagnostic accuracy and the risk of overdiagnosis.^5^ Specifically, benign prostate conditions can also elevate PSA levels, resulting in false positives that lead to excessive diagnostic interventions. Studies indicate that PSA's predictive accuracy for PCa falls below 25% when levels are under 10 ng/mL.^6^ Furthermore, PSA concentrations cannot reliably assess the risk of clinically significant PCa, necessitating histopathological confirmation for definitive diagnosis.^5^ Therefore, the identification of novel biomarkers for PCa early detection may significantly improve prognostic outcomes through enhanced diagnostic precision.

Transducin (beta)-like-1 X-linked receptor-1 (TBL1XR1) is a spermatozoa-associated protein^7^ and a subunit of the transcriptional repressor SMRT/N-CoR complex.^8^ Numerous studies have demonstrated TBL1XR1′s involvement in various malignancies, including lung, colon, ovarian, and prostate cancers. Importantly, TBL1XR1 overexpression is frequently observed in these cancers and has been linked to tumor progression, metastasis, recurrence, and poor clinical outcomes.^9^, ^10^, ^11^ In PCa, genomic gains or amplifications of TBL1XR1 correlate with higher Gleason scores and advanced tumor stages.^12^ Furthermore, TBL1XR1 expression modulates PCa sensitivity to nimotuzumab and PARP inhibitors,^13^^,^^14^ and it has been proposed as a potential prognostic biomarker across multiple solid tumors.^15^ However, the diagnostic utility of TBL1XR1 in PCa remains to be fully elucidated.

Microfibrillar-Associated Proteins (MFAPs) are extracellular matrix glycoproteins comprising five members, MFAP1 to MFAP5.^16^ MFAP5, also known as MAGP2, is a small secretory protein with a molecular weight of approximately 25 kDa that plays a key role in the structure and function of extracellular matrix microfibrils.^17^ Accumulating evidence indicates that MFAP5 is critically involved in tumorigenesis and cancer progression. It acts as an oncogene in several malignancies, including breast, head and neck, and pancreatic cancers,^18^, ^19^, ^20^ and has been shown to regulate various cellular processes such as proliferation, invasion, epithelial-mesenchymal transition, and migration. Notably, MFAP5 has been identified as a potential diagnostic biomarker in certain cancers, including gallbladder adenocarcinoma and uterine leiomyosarcoma.^21^^,^^22^ However, its role in PCa and its potential as a diagnostic marker for PCa remain largely unexplored.

In this study, we investigated the dysregulation of TBL1XR1 and MFAP5 in PCa and evaluated their diagnostic potential as well as their association with PSA. The present findings demonstrate that the combination of TBL1XR1, MFAP5, and PSA significantly enhances the accuracy of early diagnosis of PCa. This study provides a novel approach for improving the early detection of PCa.

## Materials and methods

### Ethics statement

This study was performed in line with the principles of the Declaration of Helsinki. Approval was granted by the Ethics Committee of Sanmen People's Hospital (approval number 2024-009). Written informed consent was obtained from all individual participants. This study conforms to the STROBE Statement.

### Bioinformatic analysis

The GSE229904 dataset was obtained from the Gene Expression Omnibus database (https://www.ncbi.nlm.nih.gov/geo/query/acc.cgi?acc=GSE229904). This dataset includes high-throughput sequencing data from patients with PCa. Following quality control procedures, the data were analyzed using the GEO2R online tool (https://www.ncbi.nlm.nih.gov/geo/geo2r/), an interactive web-based platform that employs the limma R package for linear modeling of microarray and RNA-seq data. Differentially expressed genes (DEGs) were identified based on statistical criteria: p < 0.05 (adjusted using the Benjamini-Hochberg method) and |log2(fold change)| > 1.

### Kyoto encyclopedia of genes and genomes (KEGG) enrichment analysis

The pathways associated with the upregulated genes were chosen for KEGG analysis using the FunRich online tool (http://www.funrich.org/).

### Clinical samples

A total of 66 patients diagnosed with PCa at the studied hospital were enrolled in this study. All cases were pathologically confirmed as PCa by biopsy. The inclusion criteria were as follows: 1) Age ≥18-years; 2) Histopathological confirmation of PCa via ultrasound-guided biopsy or radical prostatectomy; 3) Newly diagnosed with no prior anti-cancer treatment; 4) Availability of complete informed consent and baseline clinical data. Patients with any of the following conditions were excluded: 1) Urinary tract infection; 2) Prior prostate biopsy; 3) Use of 5-alpha reductase inhibitors within the past three months; 4) Recent catheterization within the past two weeks. Additionally, 60 age-matched healthy individuals without a history of cancer were recruited as the control group. Their clinical characteristics are summarized in Table 1.

**Table 1:** Clinical information of the subject.

Variables	Normal (n = 60)	Patients (n = 66)	p-value
Age, year (mean± SD or M, IQR)	64.000, 8.750	63.152±5.281	0.996
Weight, kg (mean± SD)	80.283±6.533	78.848±7.351	0.251
BMI, kg/m^2^ (mean± SD)	22.100±1.998	22.355±1.633	0.433
PSA, ng/mL (mean± SD or M, IQR)	3.151±1.567	6.465, 4.160	<0.001**
PSA ≤ 4	42 (70.00%)	4 (6.06%)	
4 < PSA ≤ 10	18 (30.00%)	55 (83.33%)	
PSA > 10	0 (0.00%)	7 (10.61%)	
fPSA/tPSA (M, IQR)	0.185, 0.070	0.120, 0.020	<0.001**
HbA1c, g/L (mean± SD)	140.483±11.820	141.773±12.555	0.555
Total testosterone, ng/mL (mean± SD)	2.022±0.266	3.926±0.593	<0.001**
Gleason grade, n (%)			
Ⅰ (Gleason score ≤6)	‒	14 (21.21%)	
Ⅱ (Gleason score 3+4)	‒	16 (24.24%)	
Ⅲ (Gleason score 4+3)	‒	20 (30.30%)	
Ⅳ (Gleason score 8)	‒	11 (16.67%)	
Ⅴ (Gleason score 9‒10)	‒	5 (7.58%)	
Stage, n (%)			
T1‒T2	‒	36 (54.55%)	
T3‒T4	‒	23 (34.85%)	
N1/M1	‒	7 (10.61%)	

### Measurement of total PSA (tPSA) and free PSA (fPSA)

Blood samples (5 mL) were collected from all participants under fasting conditions, prior to ejaculation, digital rectal examination, urinary catheterization, cystoscopy, prostate injury, prostate biopsy, or surgery. Serum was separated from the blood within 3-hours of collection by centrifugation at 3000 rpm for 20 minutes. The concentrations of tPSA and fPSA were measured using the Roche Elecsys E170 analyzer (Roche Diagnostics GmbH, Mannheim, Germany), following the manufacturer’s instructions, with corresponding commercial assay kits.

### Reverse transcription-quantitative polymerase chain reaction (RT-qPCR)

Total RNA was extracted from serum samples using TRIzol reagent (Invitrogen, Carlsbad, CA, USA). Following assessment of RNA concentration and purity, 1 μg of total RNA was reverse transcribed into complementary DNA (cDNA) using TaqMan® Reverse Transcription Reagents (Invitrogen). The mRNA expression levels were then quantified using SYBR® GreenER™ qPCR SuperMix Universal (Invitrogen) on a quantitative PCR system, and the relative expression was calculated using the 2^-ΔΔCT^ method. The primer sequences used were as follows: TBL1XR1 sense: 5’-CACCCGCTGCATTGATTTCTA-3’, anti-sense: 5’-TACGGCATCTATCAGGGACAG-3’; MFAP5 sense: 5’-GGGTCAATAGTCAACGAGGAGA-3’, anti-sense: 5’- CTGTAGCGGGATCATTCACCA-3’; GAPDH sense: 5’-ACAACTTTGGTATCGTGGAAGG-3’, anti-sense: 5’-GCCATCACGCCACAGTTTC-3’. This experiment was conducted with three biological replicates and three technical replicates. All procedures were performed by the same operator to ensure consistency.

### Statistical analysis

Data were analyzed using SPSS 25.0 software (IBM Corp., Armonk, NY, USA). The normality of baseline data was assessed using the Shapiro-Wilk test. Comparisons between two groups were performed using an unpaired Student’s *t*-test for normally distributed data, with results presented as mean ± standard deviation (SD). For non-normally distributed data, the Mann-Whitney *U* test was applied, and results were expressed as median and interquartile range (M, IQR). Categorical data were summarized as numbers and percentages [n (%)]. Univariate and multivariate logistic regression analyses were used to confirm PCa-associated factors. Receiver Operating Characteristic (ROC) curve analysis was conducted to evaluate diagnostic performance by calculating the Area Under the Curve (AUC). The Youden index was determined by the formula: sensitivity + specificity - 1. Correlation analysis was performed using the Pearson correlation coefficient. A p-value < 0.05 was considered statistically significant.

## Results

### The expression and diagnosis values of TBL1XR1 and MFAP5

**Fig. 1:**
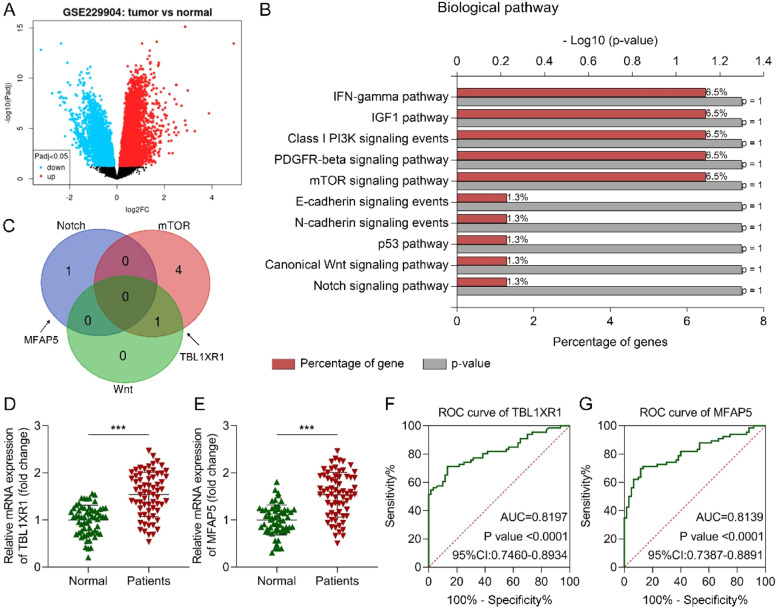
The expression and diagnosis values of TBL1XR1 and MFAP5. (A) Differentially expressed genes between PCa and normal tissues were analyzed using the GSE229904 dataset. (B) The upregulated gene-associated pathways were predicted using the KEGG enrichment analysis. (C) Venn diagram of the genes in the Notch, mTOR, and Wnt pathways. (D) TBL1XR1 and (E) MFAP5 expression in the serum of patients with PCa and healthy controls was measured using RT-qPCR. The diagnostic values of (F) TBL1XR1 and (G) MFAP5 for PCa were evaluated using the ROC curve. *** p < 0.001.

Early detection of PCa significantly reduces mortality; however, PSA exhibits notable limitations in clinical diagnosis. Novel diagnostic biomarkers are urgently required. Bioinformatic analysis was employed to identify DEGs. This dataset comprised primary tumor sequencing data from 197 PCa patients. Numerous genes were differentially expressed between PCa and normal tissues, including 259 upregulated genes (adjusted p < 0.05, |log2(fold change)| > 1; Fig. 1A). Subsequently, pathways associated with the upregulated genes were analyzed. The results indicated their involvement in multiple signaling pathways, including IFN-γ, IGF-1, Class I PI3K, PDGFR-β, mTOR, E-cadherin, N-cadherin, p53, Wnt, and Notch (Fig. 1B). Among these, proliferation-related pathways (Notch, mTOR, and Wnt) were prioritized, leading to the identification of MFAP5 and TBL1XR1 as candidate biomarkers (Fig. 1C). A total of 66 PCa patients and 60 age-matched healthy individuals were enrolled in this study (Table 1). Among the 66 PCa patients, 14 were in Gleason grade I, 16 were in Gleason grade II, 20 were in Gleason grade III, 11 were in Gleason grade IV, and 5 were in Gleason grade V. Besides, 36 patients were in stage T1‒T2, 23 patients were in stage T3‒T4, and another 7 patients were in stage N1/M1. Clinical parameters, including age, weight, BMI, and HbA1c levels, showed no significant differences between the patients and normal groups (p > 0.05). However, PSA levels, the fPSA/tPSA ratio, and total testosterone demonstrated statistically significant differences between these two groups (p < 0.001). In the univariate logistic regression analysis, high PSA and total testosterone levels were the risk factors for PCa, while the fPSA/tPSA ratio was the potential protective factor for PCa (Table 2). Furthermore, PSA was found to be an independent risk factor for PCa, and fPSA/tPSA was an independent protective factor for PCa, which was acquired from the multivariate logistic regression analysis (Table 3). Serum expression of TBL1XR1 and MFAP5 was subsequently quantified. RT-qPCR results revealed significantly elevated expression of both genes in PCa patients compared to healthy controls (both p < 0.001; Fig. 1, Fig. 1). These findings confirm their strong association with PCa progression. Diagnostic performance was evaluated using ROC curve analysis. TBL1XR1 exhibited an AUC of 0.8197 (p < 0.0001, 95% CI: 0.7460‒0.8934; Fig. 1F), while MFAP5 showed an AUC of 0.8139 (p < 0.0001, 95% CI: 0.7387‒0.8891; Fig. 1G). Collectively, these data demonstrate that elevated serum TBL1XR1 and MFAP5 levels are strongly correlated with PCa and possess significant diagnostic potential, supporting their biological relevance as candidate biomarkers for PCa detection.

**Table 2:** Univariate logistic regression analysis.

Variables	B	S.E	Wald	p-value
Age	0.02	0.031	0.411	0.521
Weight	-0.03	0.026	1.326	0.249
BMI	0.079	0.1	0.621	0.43
PSA	1.076	0.201	28.629	<0.001**
fPSA/tPSA	-79.984	15.789	25.663	<0.001**
HbA1c	0.009	0.015	0.354	0.552
Total testosterone	10.187	3.666	7.722	0.005**

**Table 3:** Multivariate logistic regression analysis.

Variables	B	S.E	Wald	p-value
PSA	4.532	1.895	5.719	0.017*
fPSA/tPSA	-223.736	89.628	6.231	0.013*

### The diagnostic value of PSA

**Fig. 2:**
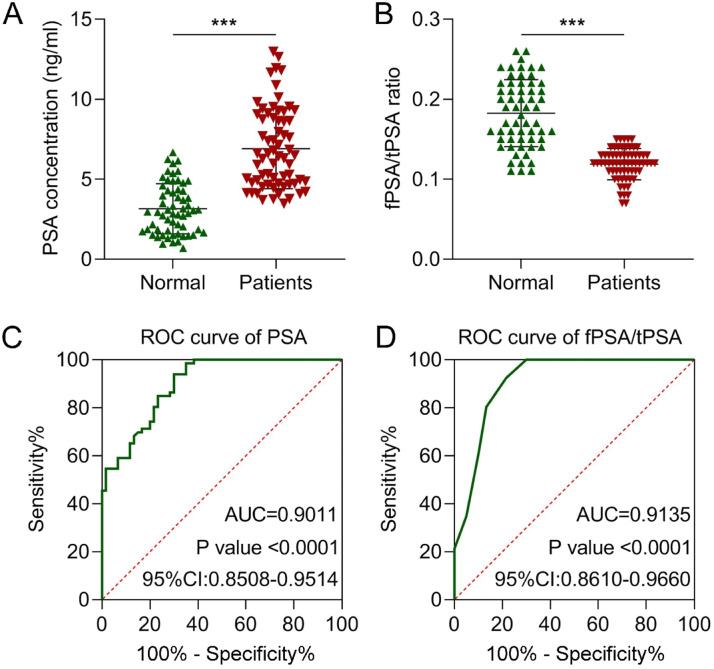
The diagnostic value of PSA. (A) PSA concentration and (B) fPSA/tPSA ratio in the serum of patients with PCa and normal subjects. The diagnostic values of (C) PSA and (D) fPSA/tPSA ratio were evaluated using the ROC curve. *** p < 0.001.

PSA is widely used for the early screening of PCa in clinical practice. Therefore, we measured serum PSA concentrations and found significantly higher levels in PCa patients compared to healthy controls (p < 0.001; Fig. 2A). The AUC of PSA was 0.9011 (p < 0.0001, 95% CI: 0.8508‒0.9514; Fig. 2C). fPSA measurement improves diagnostic accuracy when tPSA levels fall within the intermediate range of 2.5‒10 ng/mL.^23^ Consequently, the fPSA/tPSA ratio is a valuable tool for differentiating benign prostate conditions from PCa.^24^ In the present study, the fPSA/tPSA ratio was significantly lower in PCa patients (p < 0.001; Fig. 2B), with an AUC of 0.9135 (p < 0.0001, 95% CI: 0.8610‒0.9660; Fig. 2D). These results indicate that the fPSA/tPSA ratio has superior diagnostic performance compared to PSA alone.

### Correlation between PSA and TBL1XR1 or MFAP5

**Fig. 3:**
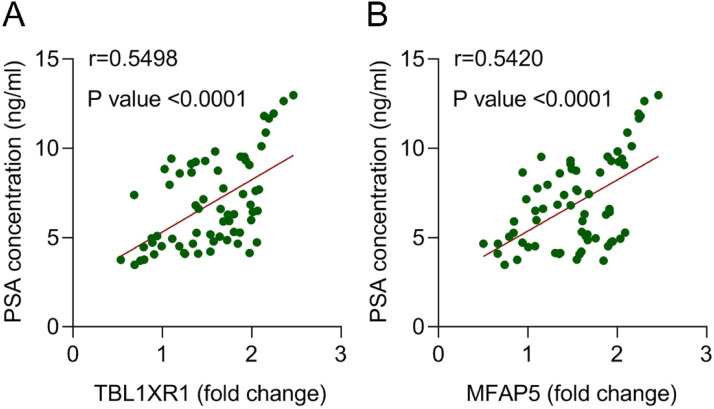
Correlation between PSA and TBL1XR1 or MFAP5. Correlation between (A) TBL1XR1 or (B) MFAP5 expression and PSA concentration in the serum of patients with PCa was evaluated using the Pearson correlation coefficient.

We next performed Pearson correlation analysis to evaluate the relationship between PSA levels and the expression of TBL1XR1 or MFAP5. The results revealed a significant positive correlation between TBL1XR1 expression and serum PSA concentration (*r* = 0.5498, p < 0.0001; Fig. 3A). Similarly, MFAP5 expression also showed a strong positive correlation with PSA levels (*r* = 0.5420, p < 0.0001; Fig. 3B). These findings indicate that both TBL1XR1 and MFAP5 are positively associated with PSA in patients with PCa, suggesting their potential synergistic roles in PCa pathogenesis. Moreover, the non-overlapping correlations support the feasibility of combining these biomarkers in clinical assays without mutual interference.

### Combination of TBL1XR1, MFAP5, and PSA improves diagnostic value

**Fig. 4:**
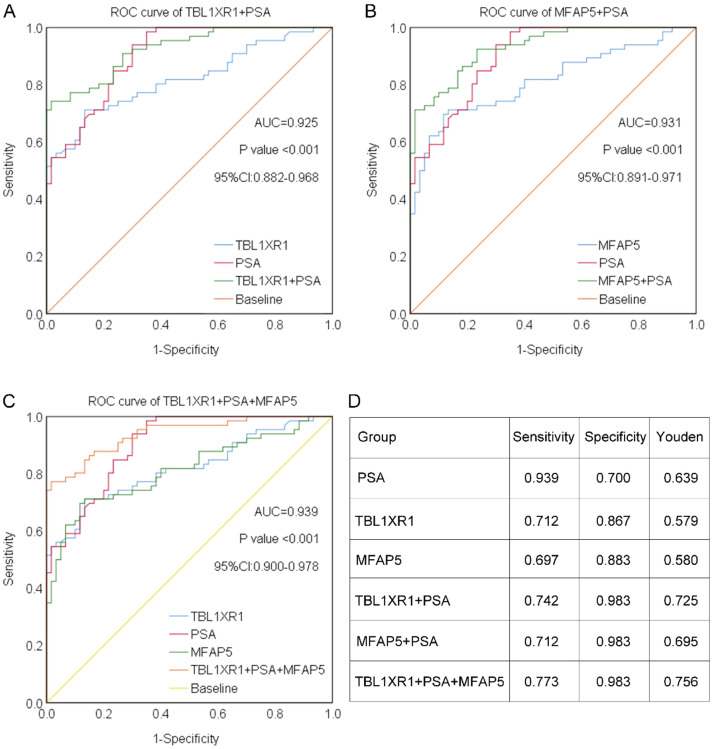
Combination of TBL1XR1, MFAP5, and PSA improves diagnostic value. (A) ROC curve of the combination of TBL1XR1 and PSA. (B) ROC curve of the combination of MFAP5 and PSA. (C) ROC curve of the combination of TBL1XR1, MFAP5, and PSA. (D) The diagnosis sensitivity, specificity, and Youden index for these biomarkers.

As previously demonstrated, TBL1XR1, MFAP5, and PSA each exhibit certain diagnostic value for PCa. To further evaluate their clinical utility, we analyzed the diagnostic performance of combined biomarkers. The AUC for the combination of TBL1XR1 and PSA was 0.925 (p < 0.001, 95% CI: 0.882‒0.968; Fig. 4A). Similarly, the combination of MFAP5 and PSA showed an AUC of 0.931 (p < 0.001, 95% CI: 0.891‒0.971; Fig. 4B). Notably, the combination of TBL1XR1, MFAP5, and PSA achieved the highest AUC of 0.939 (p < 0.001, 95% CI: 0.900‒0.978; Fig. 4C). Furthermore, sensitivity, specificity, and Youden index values are summarized in Fig. 4D. PSA alone exhibited the highest sensitivity. However, the specificity of the combinations ‒ TBL1XR1 + PSA, MFAP5 + PSA, and the three-marker panel of TBL1XR1 + MFAP5 + PSA ‒ was identical and significantly higher than that of PSA alone. Importantly, the three-marker panel yielded the highest Youden index of 0.756, indicating the optimal balance between sensitivity and specificity. Clinically, although the sensitivity of the triple-combination (77.3%) is lower than that of PSA alone (93.9%), its enhanced specificity offers a key advantage: reducing false-positive results in patients with benign conditions. This improvement addresses a major limitation of current PSA-based screening ‒ overdiagnosis and unnecessary referrals ‒ thereby enhancing the precision of PCa detection in clinical practice.

## Discussion

Screening for PCa remains controversial. PSA-based screening, although associated with reduced mortality, is also linked to overdiagnosis and overtreatment, leading to its limited adoption in most countries.^25^^,^^26^ In recent years, novel screening modalities - such as magnetic resonance imaging and targeted biopsy ‒ have been proposed to complement PSA testing, thereby mitigating PSA-related overdiagnosis.^25^ In the present study, PSA demonstrated an AUC of 0.9011, with a sensitivity of 93.9%, specificity of 70.0%, and a Youden index of 0.639. The fPSA/tPSA ratio is widely utilized as a complementary tool in PCa screening when tPSA falls within the intermediate range of 2.5‒10 ng/mL. It has been well established that assessing the fPSA/tPSA ratio enhances diagnostic specificity compared to PSA alone, significantly reducing unnecessary biopsy referrals.^25^ The present results corroborated this finding, showing an AUC of 0.9135 for the fPSA/tPSA ratio, which exceeded that of PSA alone. These data suggest that the fPSA/tPSA ratio serves as a more reliable predictor of PCa risk than PSA. However, current diagnostic approaches remain suboptimal and require further refinement. Thus, the investigation of novel biomarkers for clinical application is critical to advancing PCa screening strategies.

In this study, bioinformatic analysis was employed to identify DEGs, revealing that TBL1XR1 and MFAP5 are upregulated genes associated with cell proliferation-related pathways. Previous studies have demonstrated the critical role of TBL1XR1 in PCa progression. For instance, the miR-199a/214 cluster has been shown to target TBL1XR1, enhancing the sensitivity of PCa cells to nimotuzumab.^13^ Additionally, TBL1XR1 regulates PCa cell growth.^27^ Notably, TBL1XR1 amplification is frequently observed in aggressive PCa and is correlated with high Gleason grade and advanced disease stage, potentially driving transcriptional alterations and proliferative activity.^12^ Gu et al.^28^ further reported its diagnostic potential in pancreatic cancer, highlighting its broader relevance as a biomarker. However, the specific contribution of TBL1XR1 to PCa risk prediction remains unexplored. In the present study, serum TBL1XR1 expression was measured in PCa patients, demonstrating significantly elevated levels compared to healthy controls. ROC curve analysis revealed an AUC of 0.8197 for TBL1XR1, with a sensitivity of 71.2%, specificity of 86.7%, and a Youden index of 0.579. These findings indicate its potential utility in PCa screening. While the AUC, sensitivity, and Youden index of TBL1XR1 are lower than those of PSA alone, its higher specificity suggests a complementary role in reducing the false-positive rates associated with PSA-based screening. This improvement in specificity may address a critical limitation of current diagnostic approaches, thereby enhancing the accuracy of PCa detection.

MFAP5 functions as an oncogene involved in tumor immune regulation, metastasis, and chemosensitivity.^18^^,^^20^^,^^29^ Additionally, MFAP5 has been identified as a diagnostic biomarker for various diseases. For instance, it demonstrates potential for distinguishing uterine leiomyosarcoma from uterine leiomyoma.^30^ Notably, elevated MFAP5 expression is observed in intrahepatic cholangiocarcinoma patients, where it serves as both a diagnostic and prognostic marker.^31^ Furthermore, reduced MFAP5 levels in the stroma of gallbladder cancer have been reported, suggesting its utility in differential diagnosis.^32^ However, the role of MFAP5 in PCa remains poorly characterized. In this study, the authors found that MFAP5 was significantly upregulated in the serum of PCa patients. Its diagnostic performance was evaluated using ROC curve analysis, which yielded an AUC of 0.8139, with a sensitivity of 69.7%, specificity of 88.3%, and a Youden index of 0.580. These findings indicate that MFAP5 exhibits moderate diagnostic value, although its performance is inferior to PSA. Nevertheless, its higher specificity compared to PSA suggests potential for reducing misdiagnosis rates in PCa screening. It is worth noting that serum TBL1XR1 and MFAP5 levels were quantified via RT-qPCR in this study. While standardized protocols and quality control measures were implemented to minimize pre-analytical variability and measurement bias, inherent limitations persist. RNA instability, combined with variations in blood collection timing and sample processing procedures, may compromise the reproducibility. Additionally, technical challenges such as differential amplification efficiency and batch effects could introduce measurement errors. Addressing these pre-analytical and analytical challenges is critical before these biomarkers can be translated into routine clinical practice.

The authors further evaluated the diagnostic performance of combined biomarkers. The AUC for the combinations of MFAP5 + PSA, TBL1XR1 + PSA, and TBL1XR1 + PSA + MFAP5 were 0.931, 0.925, and 0.939, respectively, with corresponding Youden indexes of 0.695, 0.725, and 0.756. These results demonstrate that the integration of these biomarkers significantly enhances the early detection potential for PCa. Sensitivity and specificity remain critical metrics for assessing diagnostic accuracy.^33^ While PSA-based screening is associated with low specificity,^5^ these findings reveal that the combination of PSA with either MFAP5 or TBL1XR1 achieves comparable specificity to the three-marker panel (PSA + MFAP5 + TBL1XR1), but markedly higher than PSA or individual biomarkers alone. This suggests that MFAP5 and TBL1XR1 may share overlapping or complementary biological roles in PCa pathogenesis, such that pairing either with PSA already optimizes diagnostic specificity. However, the absence of further improvement with the three-marker panel implies that the diagnostic contributions of MFAP5 and TBL1XR1 may have reached a functional ceiling or are constrained by inter-marker correlations. Notably, the diagnostic sensitivity of the three-marker panel (77.3%) was significantly lower than that of PSA alone (93.9%). We hypothesize that MFAP5 and TBL1XR1, as emerging biomarkers, exhibit more specific expression patterns in PCa but may lack detectable signals at low expression levels. The inclusion of these markers in the triple-panel could impose additional constraints, potentially excluding cases positive for PSA but negative for MFAP5 or TBL1XR1, thereby reducing overall sensitivity. Despite this limitation, the enhanced specificity of the combined panels ‒ particularly the three-marker panel ‒ offers a critical clinical advantage: minimizing unnecessary biopsies and mitigating overtreatment risks associated with PSA-based screening. This trade-off between sensitivity and specificity underscores the importance of tailoring diagnostic strategies to clinical priorities. Future studies should focus on optimizing detection methods for MFAP5 and TBL1XR1 to improve their sensitivity while maintaining high specificity. Additionally, validation in larger, multi-center cohorts is essential to refine the balance between sensitivity and specificity and to establish robust clinical guidelines for integrating these biomarkers into routine PCa screening protocols.

Despite the enhanced diagnostic performance of the combination of TBL1XR1, MFAP5, and PSA, its clinical implementation faces several practical challenges. For instance, the simultaneous quantification of three biomarkers increases both operational complexity and economic cost, which may limit its feasibility in resource-limited settings. Furthermore, due to the heterogeneity of PCa and potential interactions among the biomarkers, combining multiple markers may introduce diagnostic uncertainty, complicating interpretation and potentially delaying biopsy decisions. Therefore, appropriate strategies are needed to optimize the diagnostic model, balancing accuracy, efficiency, and cost-effectiveness for broader clinical applicability.

While this study advances the identification of diagnostic markers for PCa, several limitations warrant acknowledgment. First, the relatively small sample size and single-center design may limit the generalizability and representativeness of the findings. The single-source nature of the samples could restrict the applicability of the results to broader populations. Although statistical significance was achieved (AUC = 0.939, p < 0.001), the limited sample size reduces the precision of effect estimates and increases the risk of overfitting. To address this, we plan to conduct rigorous internal and external validation studies to strengthen the robustness of our findings. Second, while the combination of PSA with MFAP5 and TBL1XR1 demonstrates improved diagnostic performance, the clinical utility of these markers requires further validation. Future research should expand the sample size and incorporate multi-center data to confirm their diagnostic value across diverse populations. Additionally, this study did not explore the biological mechanisms underlying the roles of TBL1XR1 and MFAP5 in PCa progression. Future investigations should integrate molecular biology and functional experiments to elucidate their contributions to tumorigenesis and disease advancement. Notably, the upregulation of TBL1XR1 and MFAP5 in PCa patient sera suggests their potential as oncogenic drivers. Consistent with prior reports of their roles in promoting malignancy in other cancers, the authors hypothesize that these markers similarly facilitate tumor progression in PCa. Their precise mechanisms will be a focus of future *in vitro* and *in vivo* studies, providing critical insights for clinical translation.

In conclusion, serum TBL1XR1 and MFAP5 represent promising biomarkers for PCa diagnosis, with their combination with PSA further enhancing diagnostic accuracy. While these findings are preliminary, they present a clinically actionable hypothesis: integrating TBL1XR1/MFAP5 into diagnostic panels could reduce unnecessary prostate biopsies and offer a novel approach to early PCa detection. However, external validation in larger cohorts is essential before clinical implementation. Additionally, standardizing pre-analytical protocols and evaluating real-world impact ‒ such as biopsy reduction rates and cost-effectiveness ‒ are critical steps to ensure the practical utility of these markers in routine clinical practice.

## Ethics approval and consent to participate

The study was approved by the Ethics Committee of Sanmen People's Hospital. Written informed consent was obtained from all patients. All experiments were performed in accordance with relevant guidelines and regulations.

## Consent for publication

All authors approved the final manuscript and the submission to this journal.

## Data availability statement

The data must be requested from the corresponding author.

## Authors’ contributions

XW conceived the study; XW and XL conducted the experiments; JL analyzed the data; XW was a major contributor in writing the manuscript. All authors read and approved the final manuscript.

## Funding

This study was supported by 10.13039/501100013094Social Development Project of Sanmen County in 2025 (25210).

## Declaration of competing interest

The authors declare no conflicts of interest.
